# Differential diagnosis of lipoma and atypical lipomatous tumor/well‐differentiated liposarcoma by cytological analysis

**DOI:** 10.1002/dc.24928

**Published:** 2022-01-04

**Authors:** Kana Sugiyama, Kota Washimi, Shinya Sato, Toru Hiruma, Mai Sakai, Yoichiro Okubo, Yohei Miyagi, Tomoyuki Yokose

**Affiliations:** ^1^ Department of Clinical Laboratory Kanagawa Cancer Center Yokohama Kanagawa Japan; ^2^ Department of Pathology Kanagawa Cancer Center Yokohama Kanagawa Japan; ^3^ Molecular Pathology and Genetics Division Kanagawa Cancer Center Research Institute Yokohama Kanagawa Japan; ^4^ Department of Musculoskeletal Tumor Surgery Kanagawa Cancer Center Yokohama Kanagawa Japan

**Keywords:** cytology, fluorescence in situ hybridization, lipoma, MDM2, well‐differentiated liposarcoma

## Abstract

**Background:**

Adipocytic tumors are the most common soft tissue tumors, with lipomas and atypical lipomatous tumor/well‐differentiated liposarcomas (ALT/WDL), which comprise most cases. Preoperative differential diagnosis of lipoma or ALT/WDL can provide important information for decisions regarding treatment. We evaluated the cytological findings of 20 cases of lipoma and ALT/WDL.

**Methods:**

Fluorescence in situ hybridization (FISH) was performed on formalin‐fixed paraffin‐embedded specimens (FFPE) to examine mouse double minute 2 homolog (*MDM2*) amplification in all cases. Tissue samples were collected from the center of the surgical materials, stained with Pap, and evaluated for 12 cytological parameters by six cytotechnologists.

**Results:**

The findings regarding large atypical cells, multinucleated cells, and nuclear pleomorphism were highly concordant among the cytotechnologists and were associated with *MDM*2 amplification. Large atypical cells, considered a highly specific feature of ALT/WDL, were not observed in lipoma cases. However, the sensitivity of the large atypical cell findings was not high (67%); therefore, comprehensive evaluation of multinucleated cells and pleomorphism is crucial for predicting ALT/WDL diagnosis. FISH of *MDM2* on Pap‐stained specimens was performed in four cases. In two, the results were similar to those of *MDM2* FISH performed on FFPE sections and were reproducible, whereas in the other two, the signal could not be evaluated because of the strong background coloration.

**Conclusions:**

Cytology specimens may be useful for the preoperative diagnosis of adipocytic tumors, particularly if the FISH conditions for Pap‐stained specimens and the detection accuracy of *MDM2* amplification can be improved.

## 1 INTRODUCTION

Adipocytic tumors are the most common soft tissue tumors and mostly comprise lipomas and atypical lipomatous tumor/well‐differentiated liposarcomas (ALT/WDL). ALT and WDL primarily differ on the anatomical location and resectability of the tumor. Tumors that occur in the limbs or trunk and can be resected are called ALT, while those that occur in the retroperitoneum or mediastinum and must be resected at the margins are called WDLs. The fifth edition of the WHO classification classifies ALT and WDL into the same category.^1^

ALT/WDL can become highly malignant by dedifferentiation or recurrence, thereby making it important to differentiate them from lipomas, which are benign tumors, for appropriate treatment and determination of the follow‐up period after tumor resection.^2^ The genomic abnormalities described in dedifferentiated liposarcoma support the fact that this tumor corresponds to a malignant adipocytic tumor showing progression from ALT/WDL to non‐lipogenic sarcoma of variable aspect and grade.^3^ The mouse double minute 2 homolog (MDM2) protein suppresses *TP53*, a tumor suppressor gene. Fluorescence in situ hybridization (FISH) analysis of formalin‐fixed paraffin‐embedded (FFPE) tissues has revealed *MDM2* amplification in ALT/WDL and is currently used to differentiate these tumors.^4^, ^5^, ^6^ With the development of new molecular tests showing high diagnostic specificity, fine‐needle aspiration cytology (FNAC) has gained acceptance for the preoperative assessment of soft tissue tumors.^7^ FNAC represents a versatile, low‐cost, well‐tolerated diagnostic strategy with advantages over histological biopsies.^8^ However, a detailed comparison of the cytological findings of lipoma and ALT/WDL with *MDM2* amplification has not yet been reported. In this study, we compare the cytological features of lipoma and ALT/WDL and use cytological findings for differential diagnosis. We also performed FISH to examine *MDM2* amplification in cytological specimens and evaluate the usefulness of cytology for the differential diagnosis of adipocytic tumors.

## 2 MATERIALS AND METHODS

**FIGURE 1:**
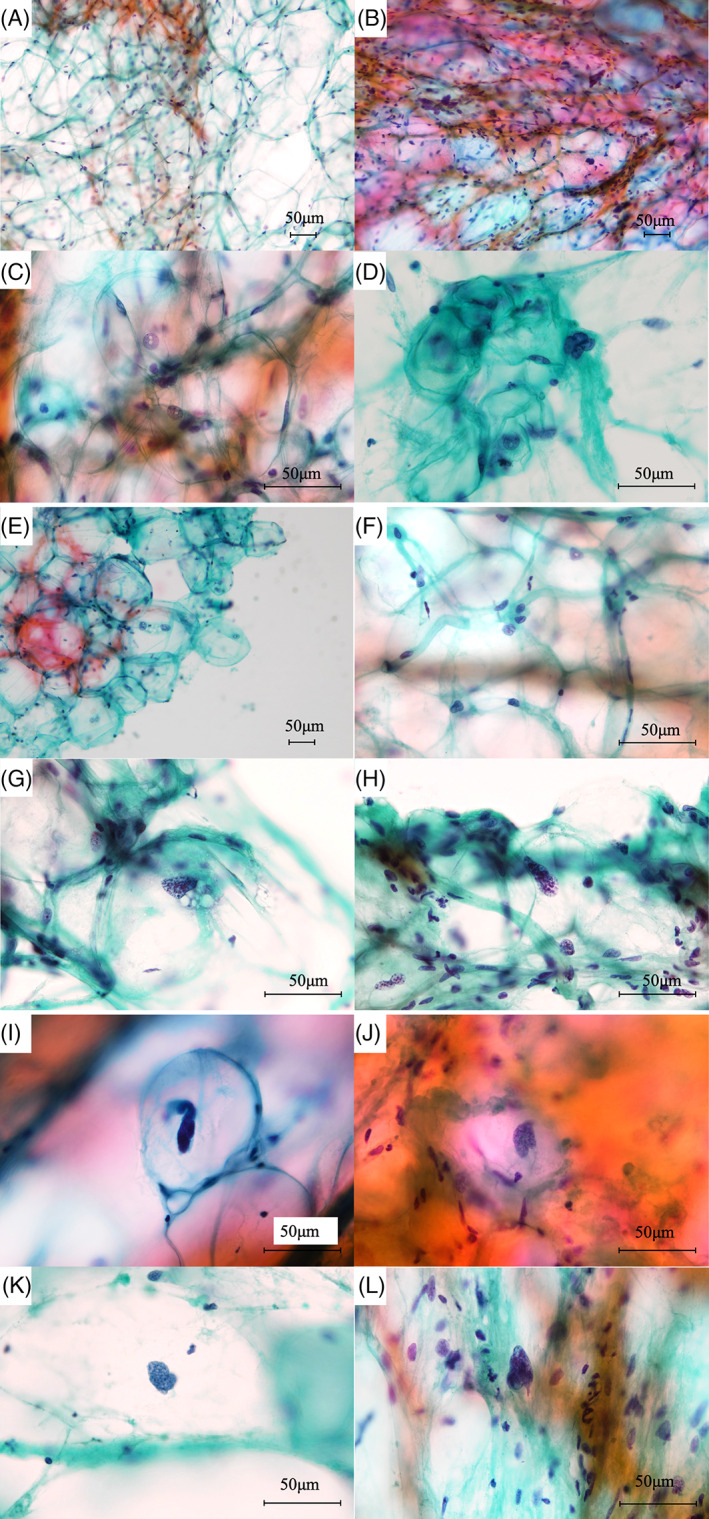
Cytological findings from Papanicolaou‐stained tissue sample. (A) The absence of nuclear pleomorphism at low magnification. (B) Prominent nuclear pleomorphism observed at low magnification. (C) Cells with intranuclear vacuoles observed at high magnification. (D) Multinucleated cells observed at high magnification. (E) Cells with nuclear enlargement and unequal nuclear size observed at low magnification. (F) Cells with nuclear enlargement and prominent nucleoli observed at high magnification. (G) A cell with hyperchromasia observed at high magnification. (H–L) Large atypical cells are defined as cells with hyperchromasia and enlarged irregular nucleus observed at high magnification. (Scale size: 50 μm) [Colour figure can be viewed at wileyonlinelibrary.com]

We reviewed the clinical and histological data of 20 patients with lipoma and ALT/WDL who had undergone resection at Kanagawa Cancer Center between 2018 and 2020. One ALT/WDL case showed partially dedifferentiated areas. We evaluated age, sex, and maximum tumor diameter as clinical parameters. Tissue samples (2–3 mm in size) were randomly collected from the center of the surgical specimens, mimicking FNAC, and subjected to Pap‐staining. If the case showed a dedifferentiated area, the sample was collected from the ALT/WDL area. Six cytotechnologists (CTs) evaluated the cell morphology, number of lipoblasts, size of adipocytes, nuclear pleomorphism, intranuclear vacuoles, multinucleated cells, nuclear enlargement, unequal size of nuclei, irregular nuclear borders, hyperchromasia, prominent nucleoli, large atypical cells, and background necrosis as a cytology routine observation. (Figure 1A–G). Large atypical cells were defined as cells with hyperchromasia and irregular nuclear enlargement (Figure 1H–l).

The morphology of each cell was evaluated on a scale of 1 to 4 (1: almost none, 2: a little, 3: common, 4: prominent), and background necrosis was assessed on a scale of 0 to 1 (0: absent, 1: present). Furthermore, each CT estimated the histological type of the lipoma or ALT/WDL based on cytological findings. More than 200 cells were examined using a WinROOF2018 image analyzer (MITANI Corporation, Tokyo, Japan), and the short nuclear diameter was measured for samples subjected to Pap staining.

The FFPE blocks of surgical samples were prepared for histological examination. The resected specimens were fixed in 10% neutral‐buffered formalin. At least one block per centimeter of the largest diameter of the tumor was prepared for histological evaluation. If the maximum diameter was 10 cm, more than 10 FFPE blocks were prepared for histological evaluation. The two pathologists evaluated the nuclear atypia of adipocytes and atypical stromal cells and made a histological diagnosis. Immunostaining for MDM2 and CDK4 and FISH examination for *MDM2* were performed in all cases using FFPE specimens. In addition, in four cases, FISH for *MDM2* was performed using Pap‐stained specimens.

The histological type determined via at least four of the six CTs was used as the cytological diagnosis result for analysis. We used the Mann–Whitney *U* test to analyze the association between clinical findings, histological diagnosis, cytological diagnosis, and length of the short diameter of the cell nuclei and the presence of *MDM2* amplification. We analyzed the association between each cytological finding and each diagnosis based on Pap‐stained specimens using Spearman's correlation and then examined the mean values. We also analyzed the association between the total score of the cytological findings of the six CTs and the presence of *MDM2* amplification using Spearman's correlation. Statistical analysis was performed using SPSS version 26 software (SPSS Inc., Chicago, IL). Statistical significance was set at *p* < .05.

### 2.1 Immunohistochemistry

Deparaffinized tumor sections were stained for CDK4 (Clone DCS‐31, Thermo Fisher Scientific, Waltham, MA) and MDM2 (Clone IF2, Thermo Fisher Scientific) using the heat‐induced epitope retrieval method. Appropriate positive and negative controls were used for all analyses. Immunostaining was evaluated based on the intensity and proportion of the tumor cells in each specimen. The intensity of staining was defined by applying Allred scoring^9^ as follows: 3+, strong; 2+, moderate; 1+, weak; −, no staining. The proportion of staining was measured for each specimen and classified by applying Allred scoring as follows: 5, > 66%; 4, 66%–33%; 3, 33%–10%; 2, 10%–1%; 1, < 1%; 0, 0%. Cases were defined as MDM2‐positive if the Allred score of the marker (defined as the combined value of the intensity score and proportion score) was more than 1. Moreover, cases were considered CDK4‐positive if the Allred score of the marker was more than 5.

### 2.2 FISH analysis

FISH for *MDM2* was performed in all 20 cases using FFPE tissues with the Vysis® LSI® MDM2 SpectrumOrange Probe (CEP® 12 [D12Z3], Abbott Molecular, Des Plaines, IL) according to the manufacturer's protocol. The probe cocktail decorates the human chromosomal region harboring *MDM2* with an orange signal and the centromeric region of chromosome 12 with a green signal. The signals were scored by counting a minimum of 20 non‐overlapping nuclei per case, and the average of *MDM2* and centromere 12 signals was calculated. An *MDM2*/chromosome 12 signal ratio of >2.0 was considered to represent *MDM2* amplification (amplification‐positive).

FISH for *MDM2* was performed using Pap‐stained specimens to evaluate the cytological findings in four cases. Using FISH in FFPE samples, two cases showed *MDM2* amplification, whereas two did not. After xylene was removed using 100% ethanol, the samples were destained with ethanol hydrochloride for 2–3 h. Then, the samples were washed with 100% ethanol and incubated overnight at room temperature (20–30°C). The specimens were immersed in 0.2% hydrochloride for 20 min, followed by immersion in distilled water for 1 min, a wash buffer for 5 min, and finally a protease solution (Abbott Molecular; pre‐warmed to 37 ± 1°C) for 10 min. The samples were again immersed in wash buffer for 5 min, and the procedure was repeated. Next, the samples were immersed in 10% neutral‐buffered formalin for 10 min, followed by immersion in wash buffer for 5 min, and the procedure was repeated. Finally, the Vysis® LSI® MDM2 SpectrumOrange Probe was added to the denatured DNA, and hybridization was carried out at 73°C for 3 min, followed by overnight incubation at 37°C. The cells were washed to eliminate nonspecific signals by immersing them in hybridization wash buffer (2X SSC/0.3% NP‐40; Abbott Molecular) preheated to 72 ± 1°C for 2 min. The specimens were immersed in wash buffer and DAPI was added, followed by observation with a fluorescence microscope Ti‐E equipped with a triple bandpass filter set, DAPI/Green/Orange v2 (Nikon Corporation, Tokyo, Japan).

## 3 RESULTS

The participants included 13 males and 7 females, with a mean age of 56 years. The most commonly affected site was the thigh (*n* = 10), and the mean maximum tumor diameter was 133 mm. Seven cases were histologically diagnosed as lipomas and 13 as ALT/WDLs. The mean nucleus short diameter of tumor cells measured from the Pap‐stained samples was 4.02 μm, with a median of 3.75 μm. The short diameter of the nucleus in most tumor cells observed in Pap‐stained samples was <5 μm, and a few tumor cells had nuclei with a short diameter of more than 10 μm. Immunostaining evaluation using FFPE tissues revealed nine MDM2‐positive cases and 11 CDK4‐positive cases. In 12 cases, *MDM2* amplification was observed by FISH on FFPE tissues (Table 1). Histologically, no necrotic findings were observed.

**TABLE 1:** Clinicopathological findings of patients with lipomas and atypical lipomatous tumor/well‐differentiated liposarcomas

			(min, max)
Male/female, *n*		13/7	
Age, years^a^		56 ± 15	(19, 90)
Tumor size, mm^a^		133 ± 73	(50, 350)
Location, n	Thigh	10	
	Neck	3	
	Head	1	
	Buttocks	1	
	Foot	1	
	Inguinal region	1	
	Lower leg	1	
	Upper arm	1	
	Retroperitoneum	1	
Histological diagnosis	Lipoma	7	
	ALT/WDL	13	
Nucleus short diameter, μm^a^	Average	4.02 ± 0.53	(3.26, 5.03)
(Papanicolaou staining)	Maximum diameter	10.07 ± 2.62	(6.21, 14.82)
Proportion of nucleus diameter, %^a^	≥5 μm	20.4 ± 10.8	(3.4, 38.2)
(Papanicolaou staining)	≥6 μm	9.6 ± 7.7	(0.5, 24.8)
	≥7 μm	4.9 ± 5.0	(0, 18.3)
	≥8 μm	2.3 ± 2.8	(0, 9.2)
	≥9 μm	1.1 ± 1.5	(0, 4.2)
	≥10 μm	0.6 ± 0.9	(0, 2.9)
*MDM2* FISH (20 cells)^b^	MDM2 signals total	168.5 (33–230)	(28, 449)
	MDM2/CEP®12 ratio	4.95 (0.98–6.70)	(0.8, 13.6)
	Amplification +/−	12/8	

Abbreviations: ALT/WDL, atypical lipomatous tumor/well‐differentiated liposarcoma; FISH, fluorescence in situ hybridization.

^a^ Values are mean ± *SD* (min, max).

^b^ Values are presented as medians (interquartile range).

In nine cases, at least four out of six CTs predicted ALT/WDL based on the cytological characteristics of Pap‐stained tissue samples. Moreover, *MDM2* amplification was observed using FISH in each of the nine cases. The nucleus short diameter was significantly longer (*p* < .001), with a larger standard deviation, in cases with *MDM2* amplification than in those without *MDM2* amplification, indicating greater variation in nuclear size. Cells in which the short diameter of the nucleus was greater than 9 μm were not observed in cases in which *MDM2* was not amplified (Table 2).

**TABLE 2:** Association between clinical findings, immunostaining results, and the length of the short diameter of cell nuclei in cases with and without *MDM2* amplification detected using FISH

		*MDM2* amplification (+)	*MDM2* amplification (−)	*p*‐value
		12 cases	8 cases	
MDM2 signal (20 cells)^a^		247.7 ± 89.6	31.9 ± 2.1	
CEP signal (20 cells)^a^		36.0 ± 8.3	33.6 ± 2.9	
MDM2 / CEP12 ratio^a^		7.24 ± 3.02	0.95 ± 0.1	
Percentage of cells with MDM2/CEP12 ratio > 2.0^a^		80.8 ± 10.4	0 ± 0	
Male/Female, n		8 / 4	5 / 3	.910
Age, years^a^		58 ± 15	51 ± 15	.427
Tumor size, mm^a^		156 ± 80	97 ± 44	.082
**Histologic specimen**				
Histological diagnosis ALT/WDL, cases		12	1	< .001
Immunohistochemistry	MDM2	9	0	.004
Positive cases^b^	CDK4	11	0	< .001
**Cytologic specimen**				
Cytological diagnosis ALT/WDL, cases^c^		9	0	.004
Nucleus short diameter, μm^a^	Average	4.32 ± 0.44	3.57 ± 0.29	< .001
	SD	1.61 ± 0.30	1.11 ± 0.15	
	Maximum diameter	11.6 ± 2.2	7.76 ± 0.87	
Proportion of nucleus diameter, %^a^	≧ 5 μm	26.4 ± 9.2	11.3 ± 5.4	.001
	≧ 6 μm	14.0 ± 7.2	3.4 ± 2.3	< .001
	≧ 7 μm	7.4 ± 5.0	1.1 ± 1.0	< .001
	≧ 8 μm	3.6 ± 3.0	0.3 ± 0.3	.001
	≧ 9 μm	1.9 ± 1.5	0 ± 0	.004
	≧ 10 μm	1.0 ± 0.9	0 ± 0	.012

Abbreviations: ALT/WDL, atypical lipomatous tumor/well‐differentiated liposarcoma; FISH, fluorescence in situ hybridization.

^a^ Values are mean ± *SD*.

^b^ Allred score MDM2: more than 1; CDK4: more than 5.

^c^ Cases in which more than half of the cytotechnologists judged ALT/WDL.

The six CTs identified the samples as lipoma or ALT/WDL based on cytological findings, with a concordance rate of 88.3%. The mean concordance rate of identifying samples as lipoma or ALT/WDL based on cytological and histopathological findings was 76.7% (65% minimum, 90% maximum), whereas that based on cytological findings and *MDM2* amplification by FISH was 78.3% (70% minimum, 85% maximum). Cytological findings with a relatively high concordance rate among CTs included lipoblasts, large atypical cells, multinucleated cells, and pleomorphism. The cytological findings that correlated significantly with *MDM2* amplification by FISH included pleomorphism, unequal size of adipocytes, irregular nuclear borders, hyperchromasia, unequal size of nuclei, nuclear enlargement, prominent nucleoli, large atypical cells, and multinucleated cells. The relationship between cytological findings and ALT/WDL identification via each CT was relatively strong for pleomorphism, nuclear enlargement, and unequal nuclear size (Table 3). In the group with *MDM2* amplification, the total scores of large atypical cells, multinucleated cells, and pleomorphism were higher than those in the group without *MDM2* amplification. The mean total score of the above three findings was less than five in the group without *MDM2* amplification, although some cases had total scores of <5 in the group with *MDM2* amplification. A single CT rescreened a 1 cm^2^ area of the Pap‐stained specimen in each case and counted the number of large atypical cells. Sixty‐seven percent (8/12 cases) Of the cases with *MDM2* amplification had large atypical cells. No large atypical cells were found in the lipoma cases (Table 4).

**TABLE 3:** Mean concordance rate between cytological findings among cytotechnologists (CTs)

	Evaluation concordance rate, %^a^		Cytological diagnosis^a^			MDM2/CEP12 ratio	
			*r*.	*p*‐value		*r*.	*p*‐value
Lipoblasts	94.9		.204	.432		.312	.181
Large atypical cells	73.4		.680	.022		.601	.005
Multinucleated cells	66.7		.641	.035		.559	.005
Pleomorphism	65.1		.812	< .001		.781	< .001
Unequal size of adipocytes	60.0		.619	.056		.711	< .001
Irregular nuclear borders	60.0		.712	.051		.694	.001
Prominent nucleoli	57.6		.593	.012		.608	.004
Unequal size of nuclear	56.6		.764	.001		.668	.001
Nuclear enlargement	55.1		.780	.001		.640	.002
Hyperchromasia	54.1		.688	.170		.693	.001
Intranuclear vacuoles	52.4		.152	.381		−.116	.625
Cytological diagnosis	88.3					.693	.001
							

*Note*: Means determined via the six CTs were used to evaluate the association between cytological findings and each individual's histological type estimated by Papanicolaou‐stained specimens. The association between the total score of cytological findings of the six CTs and the presence of *MDM2* amplification is shown.

^a^ Values are the means.

**TABLE 4:** Results of *MDM2* amplification with FISH using FFPE specimens and histological diagnosis and cytological impression

Case	FISH		Histological diagnosis	Cytological impression^a^	Cytological morphology Score average (1–4)^b^				Large atypical cell re‐examination^c^
	Number of MDM2	MDM2/CEP12			Large atypical cells	Multinucleated cells	Pleomorphism	Total score	
1	28	0.8	Lipoma	Lipoma	1.3	1.7	1.7	4.7	0
2	30	0.9	Lipoma	Lipoma	1.2	1.3	1.7	4.2	0
3	31	1.1	Lipoma	Lipoma	1.2	1.2	1.2	3.6	0
4	32	0.9	Lipoma	Lipoma	1	1.3	1.2	3.5	0
5	32	1.1	Lipoma	Lipoma	1	1	1	3	0
6	33	0.9	Lipoma	Lipoma	1	1.3	1	3.3	0
7	34	1	Liposarcoma	Lipoma	1.3	1.3	1.8	4.4	0
8	35	0.9	Lipoma	Lipoma	1.3	1.2	1.5	4	0
9	144	5.3	Liposarcoma	Liposarcoma	2.2	2.5	2.8	7.5	4
10	156	2.6	Liposarcoma	Liposarcoma	1.8	2.5	2.7	7	1
11	181	4.8	Liposarcoma	Lipoma	1	1	1.3	3.3	0
12	181	6.2	Liposarcoma	Lipoma	1	1.2	2.2	4.4	0
13	192	5.1	Liposarcoma	Liposarcoma	2.7	2.8	3.2	8.7	1
14	220	5.9	Liposarcoma	Liposarcoma	2.5	2.8	3.3	8.6	2
15	220	6.7	Liposarcoma	Liposarcoma	1.7	2.2	2.2	6.1	0
16	260	6.7	Liposarcoma	Lipoma	1.3	1.5	2	4.8	1
17	283	8.3	Liposarcoma	Liposarcoma	1.8	2.2	2.2	6.2	0
18	301	9.7	Liposarcoma	Liposarcoma	2	2.2	2.8	7	1
19	385	12	Liposarcoma	Liposarcoma	1.7	2	3	6.7	3
20	449	13.6	Liposarcoma	Liposarcoma	2.2	2.2	3	7.4	2

*Note*: Mean cytological morphology scores of the six CTs. A single CT rescreened a 1 cm^2^ area of the Papanicolaou‐stained specimen in each case and counted the number of large atypical cells.

Abbreviations: FISH, fluorescence in situ hybridization; FFPE, formalin‐fixed paraffin‐embedded specimens.

^a^ More than four out of the six CT results.

^b^ Average scores evaluated by the six CTs.

^c^ A single CT rescreened a 1 cm^2^ area of the Papanicolaou‐stained specimen in each case and counted the number of large atypical cells.

**FIGURE 2:**
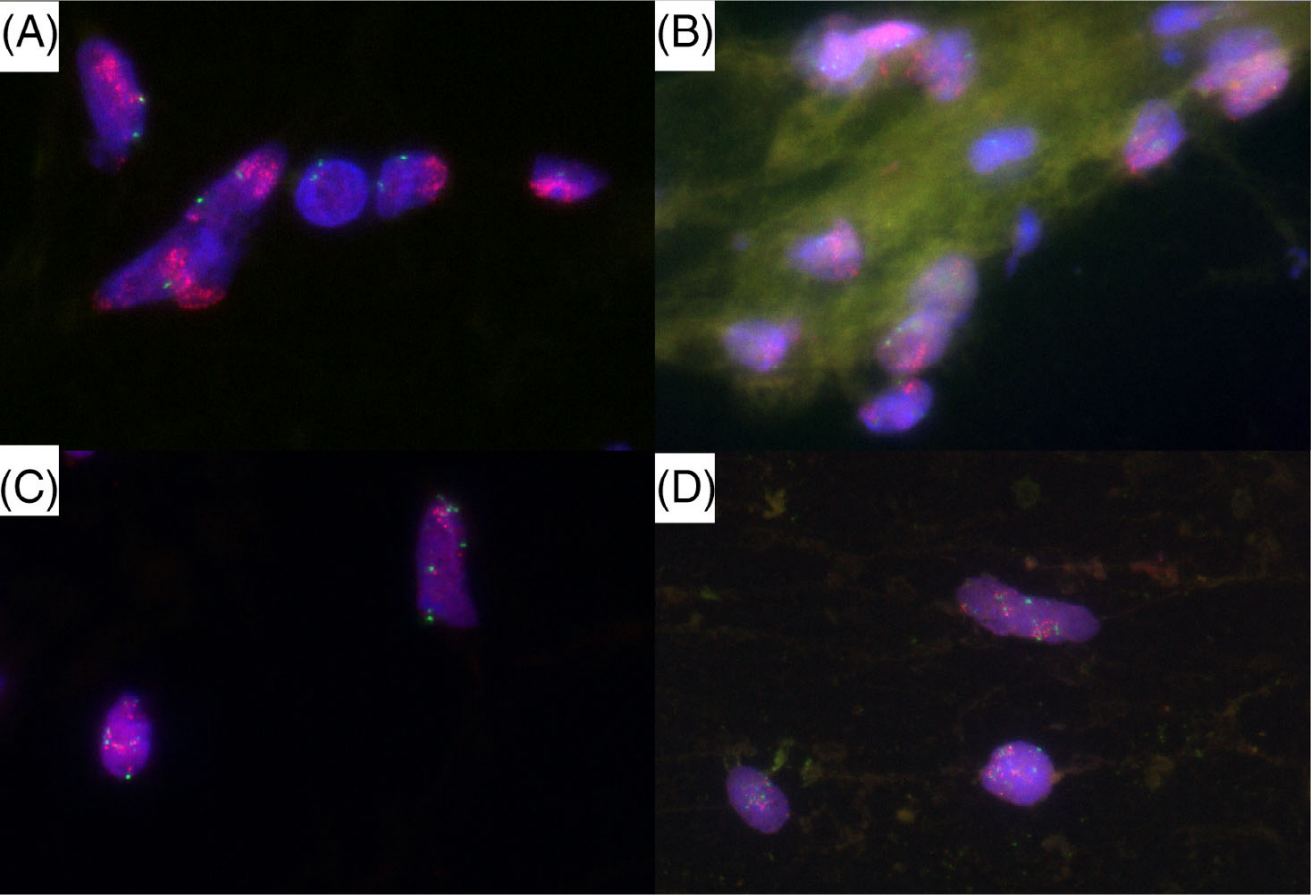
Fluorescence in situ hybridization (FISH) for *MDM2*. *MDM2* is seen as an orange signal; *CEP12* as a green signal. (A) Formalin‐fixed paraffin‐embedded (FFPE) specimen of Case 1. High level of *MDM2* signal amplification. (B) Papanicolaou‐stained specimen of Case 1. High level of *MDM2* signal amplification. (C) FFPE specimen of Case 2. Moderate level of *MDM2* signal amplification. d: Papanicolaou‐stained specimen of Case 2. Moderate level of *MDM2* signal amplification [Colour figure can be viewed at wileyonlinelibrary.com]

FISH examination was performed on Pap‐stained specimens from two cases in which *MDM2* amplification was confirmed by FISH using FFPE specimens (Table 5, Figure 2).

**TABLE 5:** Results of FISH using FFPE and Papanicolaou‐stained specimens

		MDM2^a^	CEP12^a^	MDM2 / CEP12	MDM2/CEP12 > 2.0 cells (%)
Case 1	FFPE	449	33	13.6	95
	Papanicolaou	357	41	8.3	100
Case 2	FFPE	156	61	2.6	65
	Papanicolaou	201	64	3.1	70

Abbreviations: FISH, fluorescence in situ hybridization; FFPE, formalin‐fixed paraffin‐embedded specimens.

^a^ Number of signals in 20 cells.

**FIGURE 3:**
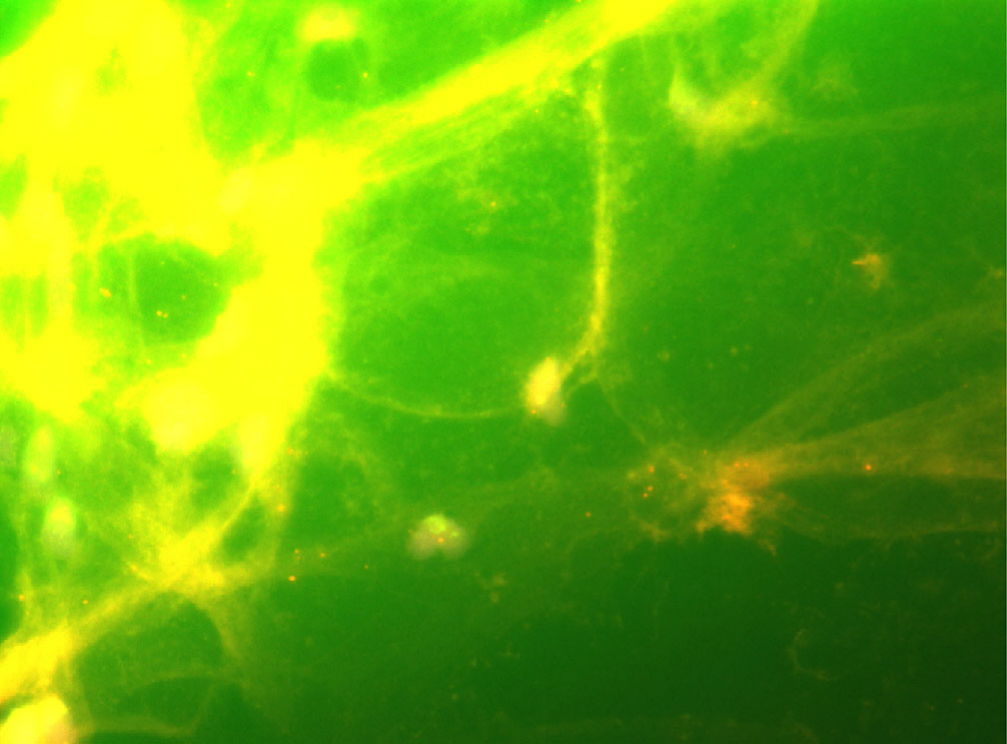
Fluorescence in situ hybridization (FISH) for *MDM2* using a Papanicolaou‐stained specimen obtained from a case that did not show *MDM2* amplification. The strong background staining made it difficult to evaluate the signal [Colour figure can be viewed at wileyonlinelibrary.com]

Case 1 exhibited relatively high *MDM2* signals in FFPE specimens, as well as higher signals in Pap‐stained specimens, whereas Case 2 exhibited relatively low *MDM2* signals in FFPE specimens and lower signals in Pap‐stained specimens. Signal evaluation by performing FISH on Pap‐stained FFPE specimens from two cases without *MDM2* amplification was difficult because of the presence of strong background signals (Figure 3).

## 4 DISCUSSION

We evaluated and compared cytological findings, *MDM2* amplification, and histological diagnosis to identify patient samples either as lipomas or ALTs/WDLs. The concordance rate for distinguishing between these conditions based on cytologic specimens and histological findings was 76.7%, indicating that small cytological specimens predicted the whole tissue histological findings with high accuracy. CT focused on pleomorphism, nuclear enlargement, and unequal size of the nucleus in predicting lipoma or ALT/WDL, but the concordance rates between CTs were low for parameters such as nuclear enlargement and nuclear size. The cytological findings with a high concordance rate among CTs were strongly associated with histology and *MDM2* amplification, multinucleated cells, pleomorphism, and large atypical cells. If the sum of the scores for the multinucleated cells, pleomorphism, and large atypical cells was 3 for lipoma and 4–12 for ALT/WDL, and the *MDM2* amplification indicated ALT/WDL, positive sensitivity was 90.3%, negative sensitivity was 64.6%, and the total concordance rate was 80.0%. In cases with no *MDM2* amplification, cases 11, 12, and 16, which had low total scores for multinucleated cells, pleomorphism, and large atypical cells in cytological specimens, were tumors with inconspicuous cellular atypia, with only a few atypical cells detected via detailed histological examination of the broad histological specimen.

Specimens from ALT/WDL cases, showing *MDM2* amplification and all three findings with score 1, showed some multinucleated cells; detailed evaluation of these findings may increase the positive sensitivity. However, few multinucleated cells were observed in lipoma cases without *MDM2* amplification. A single CT rescreened a 1‐cm^2^ area of the Pap‐stained specimen in each case and counted the number of large atypical cells. Large atypical cells were not found in the lipoma cases, and was considered a highly specific cellular finding of ALT/WDL. However, the sensitivity of the large atypical cells was not high. Comprehensive evaluation of multinucleated cells and pleomorphism is important for predicting ALT/WDL diagnosis. *MDM2* amplification may be predicted using cytological specimens with even greater probability by considering the above‐mentioned three cytological findings, namely multinucleated cells, pleomorphism, and large atypical cells.

In one case, the histological findings indicated ALT/WDL, and *MDM2* amplification was not observed using FISH. This case was diagnosed as ALT/WDL because of the presence of a small number of large nucleated cells. When identifying a distinct atypical cell is challenging, *MDM2* amplification via FISH must be confirmed for the final diagnosis. In this case, four of the six CTs presumed the sample to be a lipoma based on cytological specimens.

**FIGURE 4:**
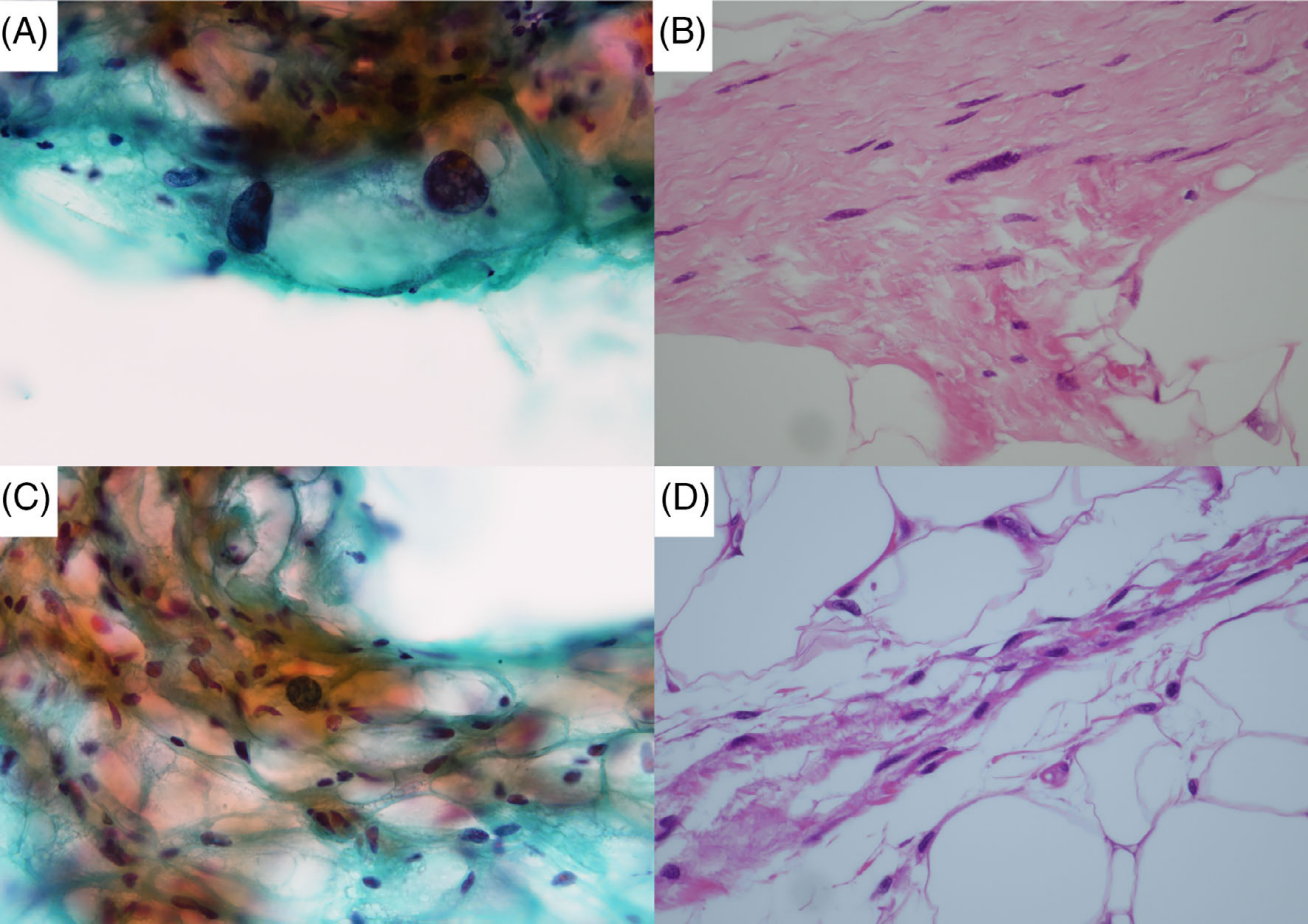
Comparison of histological images of the most conspicuous large atypical cells in all hematoxylin & eosin (HE)‐stained glass slides obtained from formalin‐fixed paraffin‐embedded specimens and the most conspicuous large atypical cells in the review of an area of about 1 cm^2^ in the Papanicolaou‐stained specimens prepared from small pieces of 2–3 mm. All figures are at the same high magnification. (A) Papanicolaou staining in Case 9. (B) HE staining in Case 9. (C) Papanicolaou staining in case 12. (D) HE staining in case 12 [Colour figure can be viewed at wileyonlinelibrary.com]

In a few cases of ALT/WDL, atypical cells were identified relatively easily in cytological specimens, even when it was difficult to identify atypical cells in the histological specimens (Figure 4).

Although tissue specimens can be examined over a wide area, cytological specimens can be used to evaluate nuclear atypia in more detail and may help improve diagnostic accuracy in combination with tissue specimens.

The length of the nucleus short diameter in the Pap‐stained cytological specimens was significantly greater in cases showing *MDM2* amplification. Additionally, a significant difference in SD values was observed, suggesting that unequal nuclear size was prominent in the *MDM2* amplification group, which may reflect pleomorphism in cytological findings. In cases without *MDM2* amplification, few cells with a nucleus short diameter > 7 μm were observed, but none of the cells had a nucleus short diameter greater than 9 μm. We did not perform immunostaining on cytological specimens since some were used for FISH examination. In contrast, we observed a significant difference between the expression of MDM2 and CDK4 as evaluated using immunostaining and the presence of *MDM2* amplification via FISH in the tissue specimens. However, we found an association between the immunostaining and FISH results only after the detailed evaluations of immunostaining and comparison of multiple cases. Immunostaining of MDM2 in ALT/WDL has been reported to be weak, localized, and not highly positive.^10^, ^11^ Therefore, immunostaining of MDM2 may not be a useful tool for the differential diagnosis of lipoma and ALT/WDL in cytology.

Although most adipocytic tumors are lipomas and ALT/WDLs, other adipocytic neoplasms should be included when performing differential diagnosis before surgery. These include adipocytic tumors, such as angiolipoma, myolipoma, chondrolipoma, spindle cell lipoma, pleomorphic lipoma, atypical spindle cell/pleomorphic lipomatous tumors, lipomatous myxoid liposarcomas, dedifferentiated liposarcomas, and pleomorphic liposarcomas, all of which were not considered in the present study. Angiolipomas are generally more common among young individuals in their late teens and early 20s.^1^ Myolipoma is a rare tumor with no atypical cells, as observed in ALT/WDL.^1^ A chondroid lipoma is a rare tumor composed of relatively cohesive clusters of mature adipocytes and variably sized lipoblasts in a chondromyxoid matrix.^12^, ^13^ Approximately 80% of spindle cell lipomas and pleomorphic lipomas arise within the subcutaneous tissue of the posterior neck, back, and shoulders.^14^, ^15^ In pleomorphic lipoma, multinucleated cells are often prominent, and may be difficult to differentiate from ATL/WDL.^1^ Atypical spindle cell/pleomorphic liposomal tumors in the hands and feet include mild to moderately atypical spindle cells, adipocytes, lipoblasts, and pleomorphic cells.^1^ These benign adipocytic neoplasms can be differentiated from ALT/WDL by examining *MDM2* amplification. Preoperative diagnosis of myxoid liposarcoma is important because preoperative radiotherapy and excision methods differ from those for ALT/WDL. Myxoid liposarcoma is more common among individuals aged 30s and 40s and tends to be less pleomorphic than ALT/WDL. Identifying *DDIT3* rearrangement using FISH break‐apart probes is a sensitive and specific strategy for the diagnosis of myxoid liposarcoma.^1^ Dedifferentiated liposarcoma often indicates ATL/WDL around the tumor, as revealed by imaging findings.^1^ Preoperative imaging findings rarely indicate pleomorphic liposarcoma as an adipocytic tumor.^16^

**FIGURE 5:**
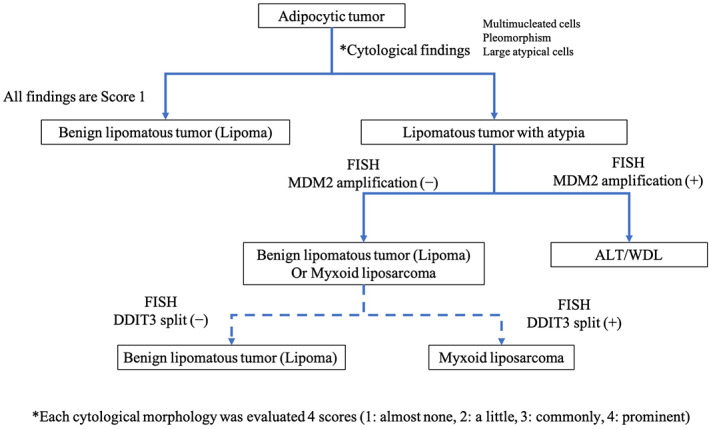
Fluorescence in situ hybridization using cytological specimens is possible with high accuracy and could be a potential strategy for diagnosing adipocytic tumors using cytological specimens. ALT/WDL, atypical lipomatous tumor/well‐differentiated liposarcomas [Colour figure can be viewed at wileyonlinelibrary.com]

Currently, while considering treatment strategies for adipocytic tumors, it is important to differentiate between benign lipomas, ALT/WDLs with the potential for recurrence and malignancy, and myxoid liposarcoma with the potential for distant metastasis that requires wide resection. FISH examination allows for differentiating between these three conditions. In the present study, the results of *MDM2* amplification obtained using FISH of cytological specimens were consistent with the FISH results obtained using FFPE specimens. In two cases with no *MDM2* amplification, it was difficult to detect signals by FISH using Pap‐stained specimens. This may be attributed to the strong background staining. To avoid background signals, it is necessary to consider methods such as dividing the sample for analysis and conducting Pap‐staining and FISH separately. It is necessary to optimize the assay conditions to improve the accuracy of *MDM2* FISH in cytological specimens of adipocytic tumors. If FISH for *DDIT3* can be performed on cytological specimens, several adipocytic tumors may be predicted from these samples (Figure 5).

It is possible to detect genes using small specimens, and minimally invasive FNAC may be used for preoperative diagnosis of various tumors, including soft tissue tumors.^5^ In the era of genetic analysis of small tissue specimens, preoperative diagnosis using FNAC, a minimally invasive test, may play a major role in determining treatment plans. Familiarity with the cell morphology of cytological specimens derived from tumors is important for making a differential diagnosis. Preoperative diagnosis of lipoma or ALT/WDL can provide important information for deciding the treatment plan. Examining the diagnostic accuracy of cytological morphology in actual aspiration biopsy specimens is essential. Given that the size of the patient data set was small (*n* = 20), we will consider a follow‐up study with a larger cohort. Our results may facilitate differential diagnoses for patients with lipoma and ALT/WDL and assist clinicians in making treatment decisions.

## 5 CONCLUSION

Lipoma or ALT/WDL can be predicted with a high probability by evaluating the cytological findings of multinucleated cells, pleomorphism, and large atypical cells in Pap‐stained tissue specimens. However, it is sometimes difficult to confirm the diagnosis based only on cell morphology, and further confirmation of *MDM2* amplification by FISH may therefore be required. With an improved accuracy of *MDM2* FISH in cytological specimens, cytology may potentially be a useful tool for the preoperative differential diagnosis of adipocytic tumors.

## CONFLICT OF INTEREST

No conflict of interest declared.

## AUTHOR CONTRIBUTIONS

Kana Sugiyama: Evaluation of cell morphology, FISH results, and immunostaining. Kota Washimi: Substantial contributions to the conception and design of the work, analysis of results, histological diagnosis, and writing of the manuscript. Shinya Sato: Analysis of clinicopathological findings. Toru Hiruma: Analysis of clinicopathological findings. Mai Sakai: Evaluation of cell morphology. Yoichiro Okubo: Analysis of clinicopathological findings. Yohei Miyagi: Analysis of clinicopathological findings. Tomoyuki Yokose: Analysis of clinicopathological findings and histological diagnosis. All authors read and approved the final manuscript.

## ETHICS STATEMENT

We applied the opt‐out method to obtain patient consent for this study. This study was approved by the Research Ethics Review Committee of the Kanagawa Cancer Center (approval no. 2020 epidemiology‐22).

## Data Availability

The data that support the findings of this study are available from the corresponding author upon reasonable request.
